# Mycorrhiza in tree diversity–ecosystem function relationships: conceptual framework and experimental implementation

**DOI:** 10.1002/ecs2.2226

**Published:** 2018-05-30

**Authors:** Olga Ferlian, Simone Cesarz, Dylan Craven, Jes Hines, Kathryn E. Barry, Helge Bruelheide, François Buscot, Sylvia Haider, Heike Heklau, Sylvie Herrmann, Paul Kühn, Ulrich Pruschitzki, Martin Schädler, Cameron Wagg, Alexandra Weigelt, Tesfaye Wubet, Nico Eisenhauer

**Affiliations:** 1 German Centre for Integrative Biodiversity Research (iDiv) Halle-Jena-Leipzig, Deutscher Platz 5e, 04103 Leipzig, Germany; 2 Institute of Biology, Leipzig University, Deutscher Platz 5e, 04103 Leipzig, Germany; 3 Department of Community Ecology, Helmholtz Centre for Environmental Research – UFZ, Theodor-Lieser-Straße 4, 06120 Halle (Saale), Germany; 4 Institute of Biology, Leipzig University, Johannisallee 21-23, 04103 Leipzig, Germany; 5 Institute of Biology/Geobotany and Botanical Garden, Martin Luther University Halle-Wittenberg, Am Kirchtor 1, 06108 Halle (Saale), Germany; 6 Department of Soil Ecology, Helmholtz Centre for Environmental Research – UFZ, Theodor-Lieser-Straße 4, 06120 Halle (Saale), Germany; 7 Department of Evolutionary Biology and Environmental Studies, University of Zürich, Winterthurerstraße 190, 8057 Zürich, Switzerland

**Keywords:** arbuscular mycorrhiza, biodiversity effects, biodiversity–ecosystem functioning, biotic interactions, ectomycorrhiza, experimental design, mutualism, MyDiv, plant–microbe interactions, resource use complementarity, species richness, tree diversity experiment

## Abstract

The widely observed positive relationship between plant diversity and ecosystem functioning is thought to be substantially driven by complementary resource use of plant species. Recent work suggests that biotic interactions among plants and between plants and soil organisms drive key aspects of resource use complementarity. Here, we provide a conceptual framework for integrating positive biotic interactions across guilds of organisms, more specifically between plants and mycorrhizal types, to explain resource use complementarity in plants and its consequences for plant competition. Our overarching hypothesis is that ecosystem functioning increases when more plant species associate with functionally dissimilar mycorrhizal fungi because differing mycorrhizal types will increase coverage of habitat space for and reduce competition among plants. We introduce a recently established field experiment (MyDiv) that uses different pools of tree species that associate with either arbuscular or ectomycorrhizal fungi to create orthogonal experimental gradients in tree species richness and mycorrhizal associations and present initial results. Finally, we discuss options for future mechanistic studies on resource use complementarity within MyDiv. We show how mycorrhizal types and biotic interactions in MyDiv can be used in the future to test novel questions regarding the mechanisms underlying biodiversity–ecosystem function relationships.

## Introduction

Concern that unprecedented rates of biodiversity change will alter ecosystem functioning and the provisioning of ecosystem services has prompted more than two decades of biodiversity–ecosystem function (BEF) research (Schulze and Mooney 1993, Loreau et al. 2001, Cardinale et al. 2012). This field of research has provided compelling evidence for a largely positive relationship between biodiversity and ecosystem functioning (Balvanera et al. 2006, Cardinale et al. 2012) in controlled experiments as well as in nature (Hautier et al. 2014, Duffy et al. 2017). Despite this emerging consensus regarding the significant role of biodiversity for ecosystem functioning, the underlying mechanisms driving this relationship are still not well understood.

Theory predicts that positive plant diversity effects on ecosystem functioning should arise if intraspecific competition in communities is higher than interspecific competition (Loreau and Hector 2001). As a consequence, plant traits related to resource use may be particularly influential drivers of competitive interactions in plant communities. If species are dissimilar in their resource use strategies, they avoid competition for limiting resources (hereafter resource use complementarity). This reduction in interspecific competition should provide higher levels of ecosystem functioning than a community of species with more similar resource use strategies (Heemsbergen et al. 2004, Jousset et al. 2011). For example, species asynchrony (which may indicate resource use complementarity over time; de Mazancourt et al. 2013, Hautier et al. 2014) and spatial dissimilarity in light use in tree crowns (resource use complementarity in space; Williams et al. 2017) have been suggested as significant biological mechanisms that underlie positive BEF relationships. Consequently, much effort has been placed to identify species traits that are essential drivers of BEF relationships (Ebeling et al. 2014, Tobner et al. 2014).

Resource use complementarity among species also relies on biotic interactions across guilds of organisms (Eisenhauer 2012, Hines et al. 2015). In plant communities, the acquisition of soil nutrients is not only a function of rooting depth (Mueller et al. 2013, Oram et al. 2017) and root traits, but requires interaction partners like mycorrhizal fungi (de Kroon et al. 2012). Plant mycorrhization occurs in most of the terrestrial plant species and is commonly known to be beneficial to plants by enhancing their growth (e.g., Smith and Read 2010, van der Heijden et al. 2015). Mycorrhizal fungi supply plants with water and nutrients in exchange for photosynthates and therefore co-determine the outcome of plant competition (Fitter 1977, Zobel and Moora 1995, van der Heijden et al. 1998, Scheublin et al. 2007, Wagg et al. 2011*a*, *b*, Merrild et al. 2013). In fact, mycorrhizal taxa themselves have evolved ways to reduce competition in space and time and possess traits as various as the plants with which they associate (Koide 2000, Smith et al. 2000, Jansa et al. 2005, van der Heijden and Scheublin 2007, Thonar et al. 2010). As a consequence, mycorrhizal fungi are thought to play a critical role in the maintenance of plant diversity (Francis and Read 1994, 1995, van der Heijden et al. 1998) and positive BEF relationships (Klironomos et al. 2000, Schnitzer et al. 2011, Eisenhauer 2012). However, mycorrhizal associations are not beneficial in all cases. They form a continuum from being beneficial to being detrimental that depends on factors like environmental conditions and the developmental state of the associations (Johnson et al. 1997).

For mycorrhizal associations to maintain plant diversity and improve ecosystem function, the presence and diversity of fungal associations should increase resource partitioning among the different plant species with which they associate (Klironomos et al. 2000, Bever et al. 2010, Wagg et al. 2015). This may be true for different plant species associating with different mycorrhizal fungal species and also with different mycorrhizal types. Mycorrhizal types considerably differ in their morphological and physiological traits that facilitate dissimilar soil nutrient uptake processes. Several studies have shown the significance of arbuscular mycorrhizal fungal (AMF) species diversity for plant performance (Vogelsang et al. 2006, Maherali and Klironomos 2007, Wagg et al. 2011*a*, *b*, 2015, Reinhart and Anacker 2014). However, including both mycorrhizal types as potential biotic interactions driving resource use complementarity in studies is crucial as they typically co-occur in natural ecosystems.

Our paper provides a conceptual framework for including positive biotic interactions across guilds of organisms—more specifically between plants and mycorrhizal types—to study potential mechanisms behind resource use complementarity of plants as well as the consequences for plant competition and BEF relationships. First, we provide an overview of the current understanding of the effects of biotic interactions on resource use complementarity and how this might enhance ecosystem function. Second, we highlight the urgent need for including plant–mycorrhiza interactions in studies to deepen the mechanistic understanding of resource use complementarity. Third, we introduce a recently established field experiment that utilizes this conceptual framework. The study uses different pools of tree species that associate with dissimilar types of mycorrhizal fungi to create experimental gradients in tree species richness. The different experimental combinations between tree species and mycorrhizal fungi span a hypothesized gradient in coverage of resource niche space and thus provide a predictive framework for resource use complementarity. Fourth, we provide an outlook of potential future studies in this experimental setup that may advance the mechanistic understanding of BEF relationships.

## Biotic Interactions Influence Resource Use Complementarity and Ecosystem Functioning

Resource use complementarity is the most commonly cited ecological mechanism for driving enhanced ecosystem functioning in diverse systems (reviewed by Cardinale et al. 2006, see also Hooper et al. 2012, Scherer-Lorenzen 2014, Tilman et al. 2014). However, empirical evidence for resource complementarity has been based primarily on plant–plant interaction studies (Eisenhauer 2012, Williams et al. 2017). For example, belowground studies demonstrate that when plant roots grow deeper in mixture (an indicator of resource partitioning), ecosystem functioning is enhanced (Mueller et al. 2013, Oram et al. 2017, but see Ravenek et al. 2014).

Biotic interactions can enlarge biotope space (Hutchinson 1978, Dimitrakopoulos and Schmid 2004) and enable facilitation among plant species and plant functional groups (Ebeling et al. 2014). In general, there is evidence that positive biotic interactions may increase resource supply to plants, for example, by mediating the effects of nitrogen-fixing plants. Nitrogen is considered the most limiting nutrient in many plant communities (Klapp 1971, Ellenberg 1977, Vitousek and Howarth 1991). Some plants are able to avoid nitrogen limitation by hosting nitrogen-fixing rhizobacteria. The presence of nitrogen-fixing plants increases the overall availability of nitrogen for the community (Fargione et al. 2007, Temperton et al. 2007, Gubsch et al. 2011). Yet, the community performance is higher with nitrogen-fixing plants (Eisenhauer 2012). However, study results are sometimes conflicting on the potential importance of resource use complementarity for enhanced ecosystem function. In a depth-controlled tracer experiment in a savanna, Kulmatiski et al. (2010) found small differences between grass and tree water uptake across seasons, while other studies observed significant differences in the vertical and horizontal distribution of nutrient uptake (Kahmen et al. 2006 for grasslands, Kulmatiski and Beard 2013).

In this paper, we focus on interactions among different plant species and between plants and soil organisms as those may particularly be promising to explain plant diversity–ecosystem function relationships (Eisenhauer 2012, Kulmatiski et al. 2012, Connolly et al. 2013). According to Eisenhauer (2012), there are four main ways in which plant species may engage in aboveground–belowground biotic interactions to alter resource use complementarity and thereby enhance ecosystem function: (1) enlarging biotope space, (2) mediating effects of nitrogen-fixing plants, (3) increasing plant community resistance to antagonists, and (4) maintaining plant diversity. The link between biotic interactions and resource use complementarity may be determined by both positive and negative biotic interactions (e.g., Schnitzer et al. 2011). However, the role of positive interaction partners in resource use complementarity has been underestimated by the BEF literature, despite the ability of positive interactions to meet all of the four criteria listed above (Wright et al. 2017). While Eisenhauer (2012) reviewed how plant resource use complementarity can be influenced by a multitude of aboveground-belowground biotic interactions, we here focus specifically on interactions between plants and mycorrhizal fungi in the next sections due to the significant role of mycorrhiza for plant nutrition, competition, fitness, and resistance to plant antagonists.

## Mycorrhiza and Their Role in Bef Relationships

Mycorrhiza is a symbiosis that has evolved between a vast diversity of terrestrial plants and soil fungi, where fungi acquire plant photosynthetic carbon and in exchange provide the plant host with enhanced uptake of soil nutrients (Smith and Read 2010). These associations are thought to have facilitated the radiation of terrestrial plants over 400 million years ago (Remy et al. 1994, Brundrett 2002). Plants and soil fungi have since evolved different types of mycorrhiza that vary substantially in their life strategies and thus the mechanisms by which the fungal partners provide soil resources to their plant hosts (Peterson et al. 2004, Johnson et al. 2012, 2017). Arbuscular mycorrhizal fungi are the oldest and most abundant monophyletic fungal phylum (Glomeromycota) that obtain carbon exclusively from their host plants and form obligate associations with around 80% of the land plants (Brundrett 2009). The primary function of AMF for their plant host is the provisioning of soil phosphorus that would otherwise be inaccessible to the plant host (Smith and Read 2010). The exchange of phosphorus for carbon in this endophytic mycorrhizal type occurs within the inner cortical cells of the plant host fine roots (Peterson et al. 2004). A second type, the ectomycorrhiza, evolved repeatedly within diverse fungal saprotroph phyla in the Asco- and Basidiomycota (Read and Perez-Moreno 2003, Bruns and Shefferson 2004). These ectomycorrhizal fungi (EMF) typically invest more into forming their mycorrhizal association, relative to AMF, by the development of a mantle (tightly woven sheath of hyphae) around root tips. Nutrient exchange with their host occurs through a hartig net (highly folded hyphal structures) that forms around epidermal and inner cortical cells of root tips. The development of EMF associations is generally a slower process, and the mycorrhizal structures have a lower turnover rate than AMF associations (Chilvers et al. 1987, Comas and Eissenstat 2009). These fungi can mobilize both organic and mineral plant resources from diverse substrates, and thus, some are not obligate mycorrhizal fungi (Peterson et al. 2004, Plett and Martin 2011).

Arbuscular mycorrhizal fungi and EMF coexist in soil and are able to build huge hyphal networks for nutrient acquisition that may interconnect various plant species (Leake et al. 2004, Simard and Durall 2004, Horton 2015). The extent of plant–fungal interactions is known to depend on the identity and diversity of the plant host and fungal partner, as well as on the abiotic context, for example, resource availability (Johnson et al. 2010, Smith and Read 2010). Arbuscular mycorrhizal fungal species differ in particular characteristics and strategies that benefit plants, such as pathogen resistance/defense strategies (Gange and West 1994, Newsham et al. 1995, Azcón-Aguilar and Barea 1997, Powell et al. 2009, Sikes et al. 2009) and strategies in resource acquisition from soil (Smith et al. 2000, Jansa et al. 2005, Thonar et al. 2011). Ectomycorrhizal fungi also have a vast diversity of morphological and growth characteristics by which they are thought to have evolved foraging strategies (Agerer 2001, 2006, Tedersoo and Smith 2013). Resource acquisition by mycorrhizal fungi often targets plant-unavailable or limiting resources, such as phosphorus (Jeffries et al. 2003). The transfer of these limiting resources to plant hosts may occur via fungal mineralization and direct nutrient supply to the host or by the translocation of resources among plants via the hyphal networks interconnecting them (Selosse et al. 2006, van der Heijden and Horton 2009, Johnson et al. 2012). Here, we focus only on the direct nutrient supply to the host.

Plant species may host different communities of mycorrhizal fungi that, consequently, allocate different sets of resources to the plant species. For instance, different AMF species may acquire phosphorus from different locations in the soil or through their temporal activity pattern (Jansa et al. 2005, Lindahl et al. 2007, Oehl et al. 2009, Thonar et al. 2010, Dumbrell et al. 2011). Consequently, a higher AMF species richness may foster a more comprehensive resource uptake. It has been noted that the identity of a particularly effective mycorrhizal taxon may result in similar effects as a diverse mixture of AMF (Vogelsang et al. 2006, Wagg et al. 2011*b*). However, Wagg et al. (2011*b*) documented that the importance of single fungal species in a diverse AMF community may be altered by environmental conditions. This could suggest that a more diverse composition of mycorrhiza may buffer the functioning of the mycorrhizal community throughout short-term (pulse) disturbances, whereas it was shown that repeated stress (press disturbances) decreases AMF species diversity (Millar and Bennett 2016). Further, the functional characteristics of AMF are thought to be phylogenetically conserved (Powell et al. 2009), and it has been demonstrated that more phylogenetically dispersed AMF communities can enhance ecosystem functioning (Maherali and Klironomos 2007).

Positive effects of EMF richness on plant nutrient uptake and growth have also been observed (Baxter and Dighton 2001). These studies lend support to the concept that more functionally diverse mycorrhizal fungal communities may contribute to maintaining a more diverse plant community by enhancing the access and use of the available resource pool to plants resulting in relaxed plant–plant competition for soil resources (van der Heijden et al. 1998, Klironomos et al. 2000, Scheublin et al. 2007, Jansa et al. 2008, Wagg et al. 2011*b*, 2015). Thus, it is conceivable that the presence of two distinct mycorrhiza types (AMF and EMF), that have even more distinct life style and foraging strategies, may have important implications for resource partitioning among their associated plant hosts in addition to the differences between plant species per se. Importantly, the two mycorrhizal types are known to further indirectly affect plant performance through various mechanisms which may add to or dilute the effects of resource use complementarity on the positive biodiversity–ecosystem functioning relationship. For instance, Bennett et al. (2017) found positive plant–soil feedback effects in EMF-trees favoring conspecific plant individuals, whereas they found negative plant–soil feedback effects in AMF-trees favoring heterospecific plant individuals, with the latter potentially increasing ecosystem functioning. However, in this paper, we focus on the direct effects only.

Associations with different mycorrhizal types may therefore increase resource use complementarity among plant species in diverse plant communities. It has recently been shown that AMF- and EMF-trees may differ in their soil resource foraging strategies due to their rooting characteristics and associations with their fungal partners (Chen et al. 2016). To date, however, few studies using both mycorrhizal types have focused on the spectra of resource uptake strategies among plant species. To disentangle the effects of mycorrhizal types from that of plant species identity and other abiotic and biotic interactions, it is crucial to manipulate mycorrhizal types along a plant diversity gradient in experimental studies as has been done for AMF species in grassland mesocosms (Wagg et al. 2011*a*, *b*, 2015). Tree diversity experiments provide the advantage of a more balanced ratio of AMF- and EMF-associated species compared to grasslands, where nearly all plant species present do not associate with EMF (Smith and Read 2010).

**Fig. 1:**
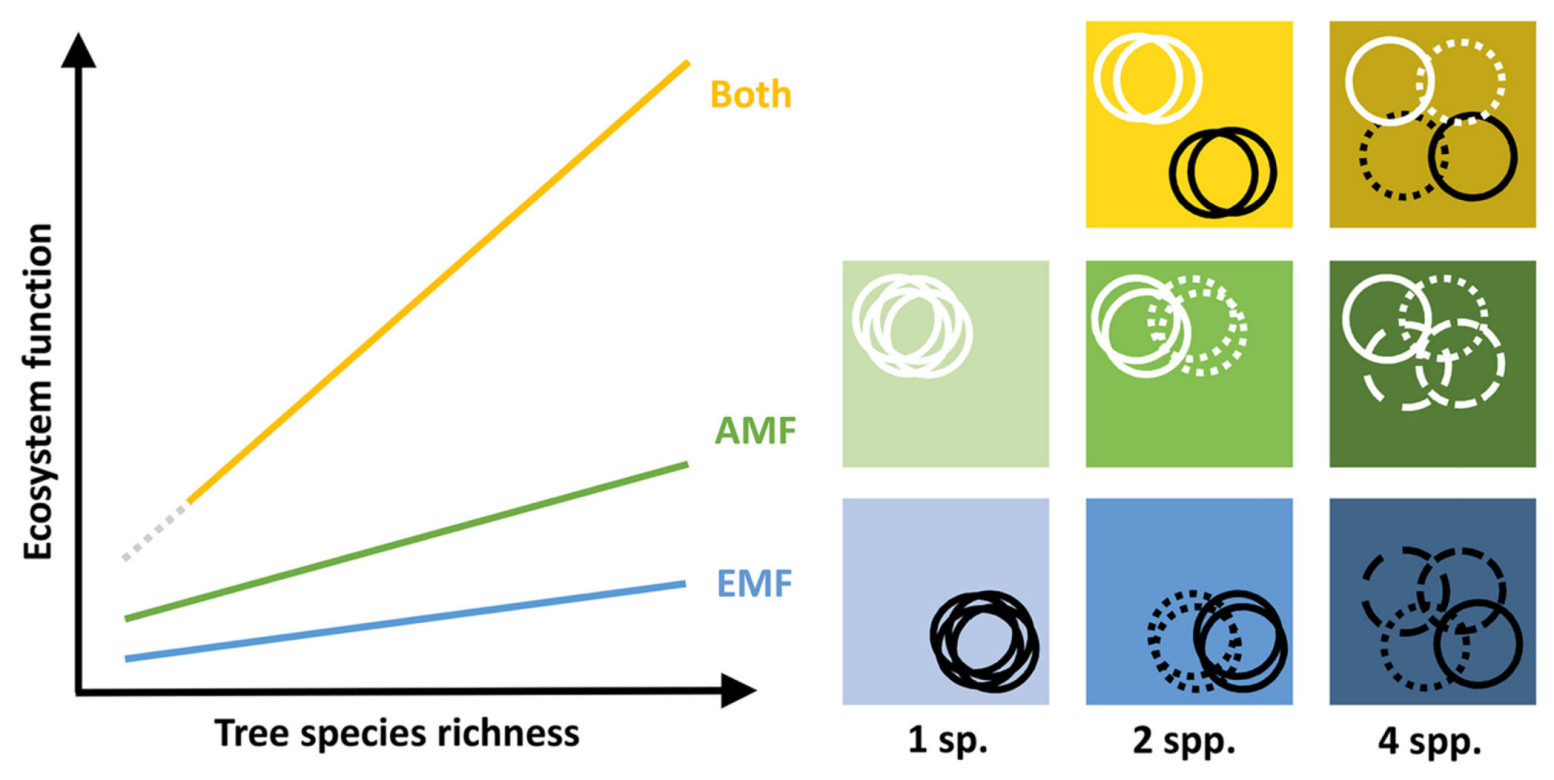
Conceptual figure illustrating (left) the main hypothesis and (right) the underlying resource use scenarios in tree species that coexist in a community and are limited by a set of resources in MyDiv (modified from Klironomos et al. 2000). We assume that the positive relationship between tree species richness and ecosystem functioning will differ among tree communities. Communities of only arbuscular mycorrhiza-associated tree species (AMF) will have higher ecosystem functioning and show stronger tree diversity effects on ecosystem functioning compared to only ectomycorrhiza-associated tree species (EMF) as indicated by intercept and slope of the graphs, respectively. The soil at the experimental site is nitrogen-rich and presumably phosphorus-limited favoring AMF-tree species performance as AMF are assumed to supply plants more efficiently with phosphorus. Accordingly, the resource space (represented by the colored boxes) occupied by the tree species (represented by circles with different line types; black circles represent EMF-species, white circles represent AMF species) in each community differs as indicated by different positions of the circles within the boxes. We expect that ecosystem functioning will be highest at the highest tree diversity level in tree communities associated with both mycorrhizal types (Both, dark yellow box). In such tree communities, resource use complementarity should be highest as indicated by the lowest level of overlap among circles (low competition for the same resources) and the highest exploitation of the available resource space. This is expected to result in the highest performance of the tree community.

In a new field experiment called MyDiv, we aim to address gaps in the understanding of contributions of mycorrhizal types and their respective fungal communities to resource use complementarity in plant communities. The experiment was set up to test the following main hypothesis: Tree communities with diverse mycorrhizal types will utilize soil nutrients more complementarily than tree communities with a single mycorrhizal type. Therefore, treatments combining high tree species richness and presence of both mycorrhizal types are expected to increase resource uptake and, consequently, complementarity resulting in the highest tree performance (Fig. 1).

## Investigating Links between Biodiversity and Ecosystem Functioning with Trees and Mycorrhizal Fungi

Biodiversity–ecosystem function relationships have been mostly studied in grasslands, but more recently, tree experiments in different biomes confirm positive relationships (e.g., Liang et al. 2016). Tree diversity experiments can significantly contribute to the understanding of resource use complementarity in diverse plant communities. They allow for studying performance and biotic interactions within plant communities on the individual, neighborhood, and plot scale (van der Plas et al. 2016, Grossman et al. 2017). In addition, measurements of physiological processes in plant individuals in situ reflect how individuals are responding to their surrounding community and are relatively straightforward. Tree phytometers enable highly standardized measurements on physiological processes and, thus, a fine resolution of data. Both approaches are essential for investigating plant–plant as well as plant–fungal interactions, in order to better integrate biotic interactions across guilds of organisms into BEF relationships (e.g., Thompson et al. 2012, Soliveres et al. 2016, Eisenhauer 2017) and to consider different ecological scales in BEF research (Cardinale et al. 2012, Isbell et al. 2017).

To better understand the role of mycorrhizal associations in resource use complementarity and the contributions of plant–fungal interactions to species coexistence in plant communities, it is crucial to study those interactions under field conditions. Combining the use of tree experiments with manipulations of plant traits related to resource acquisition, such as mycorrhizal types, enables the evaluation of how identity and diversity of plant interaction partners may alter interspecific competition and, consequently, influence complementary resource use within plant communities.

We set up a new tree diversity experiment called “MyDiv.” The abbreviation stands for The Role of Mycorrhiza in tree Diversity effects on ecosystem functioning. This experiment manipulates the two main mycorrhizal types (via respective tree species selection) along a tree species richness gradient comprising monocultures, two-species and four-species mixtures. The mycorrhizal treatment is comprised of tree communities that, according to literature, predominantly associate with AMF, EMF, or mixtures of tree species that associate with either one of those mycorrhizal types. In the following, we introduce the site, the design, and basal measurements of the recently established MyDiv experiment as well as show initial results of biodiversity effects on productivity. In the future, MyDiv can gain from the integration of other projects, such as the oak phytometer project (PhytOakmeter; Herrmann et al. 2016) and the global network on tree diversity experiments TreeDivNet (Verheyen et al. 2016) outlined below, that allow to address more specific or even broader questions.

### Site

**Fig. 2:**
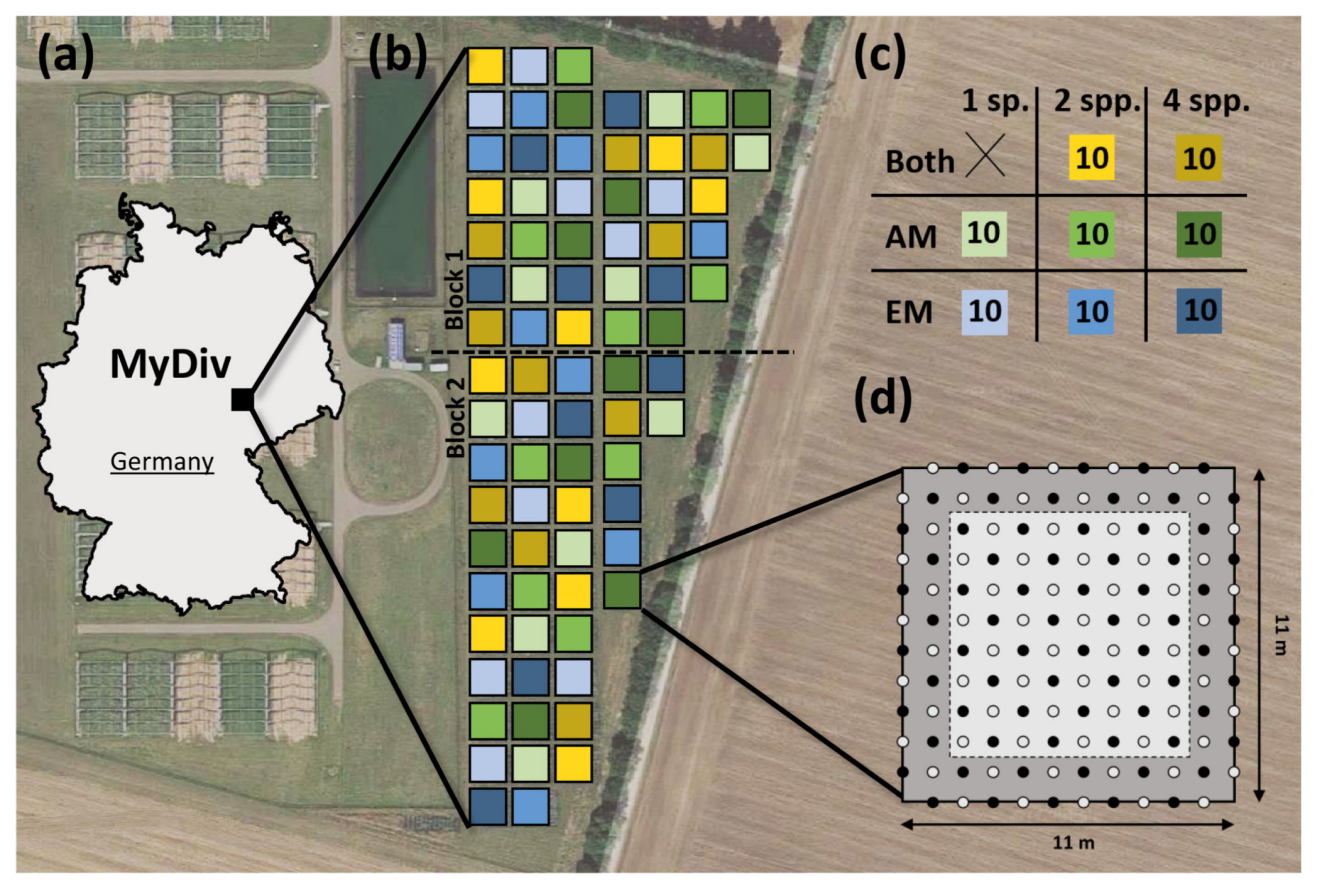
(a) Map of Germany with the location of MyDiv (latitude, 51°23′ N, longitude, 11°53′ E), (b) the within-site experimental design, (c) an overview of the main treatments with respective color coding for (b), and (d) the within-plot experimental design. Numbers in boxes indicate the number of replicates. AMF, arbuscular mycorrhizal fungi; EMF, ectomycorrhizal fungi; Both, both mycorrhizal types. For details on replication and species composition of the plots, see Appendix S1: Fig. S4. Aerial background photography: Imagery 2017 Google, Map data 2017 GeoBasis-DE/BKG (2009), Google.

The MyDiv experiment is located in Saxony-Anhalt, Germany, southwest of Halle (51°23′ N, 11°53′ E) at the Bad Lauchstädt Experimental Research Station of the Helmholtz Centre for Environmental Research–UFZ (Fig. 2a). The site is located at 114–116 m a.s.l. and is characterized by a continental summer-dry climate with a mean annual precipitation of 484 mm and a mean annual temperature of 8.8°C (Altermann et al. 2005). The parent material is silt over calcareous silt (loess), and the soil type is classified as Haplic Chernozem developed from loess with silt loam texture. Chernozem soils are very fertile and characterized by a thick humus horizon with a stable aggregate structure, a high base saturation, high water-retention capacity, and high bioturbation rates (Altermann et al. 2005). The soil consists of an Ap horizon down to 30 cm depth followed by an Ah horizon at a depth of 30–45 cm and a C horizon starting at 45 cm depth (Altermann et al. 2005).

Most of the characteristics of the upper soil of the site (0–10 cm depth), namely inorganic and organic carbon, total nitrogen, and total phosphorus concentrations, pH, soil texture, and microbial properties, show a gradient along the north–south axis of the site (Appendix S1: Figs. S1 and S2). The elemental concentrations and the proportion of sand generally decrease along this gradient (C_inorg_: 0.26– 0.03%, C_org_: 2.37–1.63%, N_tot_: 0.21–0.14%, P_tot_: 690–400 mg/kg, sand: 6.7–5.2%), whereas the proportion of silt increases (65.7–76.5%). Microbial basal respiration (BAS) and biomass carbon (C_mic_) decrease along the north–south axis of the site (BAS: 3.79–0.56 μL O_2_·h^−1^·[g soil DW]^−1^, C_mic_: 737.27–166.69 μg C_mic_/g soil DW). The proportion of clay, pH, and fungal and bacterial biomass do not show any consistent spatial pattern. The natural vegetation of this area is mixed broad-leaved forest, but the area has been converted to agricultural land since the beginning of human settlement due to the high fertility of this soil type. The site had been used for agriculture until 2012 at which point it was converted to a grassland for two years until being plowed to prepare the site for the establishment of MyDiv.

### Tree species selection

To study the effects of mycorrhizal type on the relationship between tree species richness and ecosystem functioning, we established a gradient in tree species richness comprising monocultures, two- and four-species mixtures (Fig. 2c; Appendix S1: Fig. S5). In addition, we set up a mycorrhizal type treatment with three levels comprising only AMF-trees, only EMF-trees, and AMF- and EMF-trees in mixture. Several deciduous tree species, such as in the genus *Populus*, are known to associate with both mycorrhizal types (Harley and Harley 1987*a*). Multiple associations may occur simultaneously, depending on environmental conditions or nutritional status of the plant, or occur at different stages of root development and plant growth (Chen et al. 2000). However, patterns and mechanisms driving the establishment of mycorrhiza are still not fully understood and, thus, are poorly predictable or open to experimental manipulation. In MyDiv, we refrained from using tree species that commonly associate with both mycorrhizal types to not confound the experimental design. Our mycorrhizal treatment was established by using tree species that, based on an extensive literature review (e.g., Wang and Qiu 2006), mostly associate with only AMF or EMF. Therefore, tree species identity is nested in mycorrhizal type identity.

For species selection, a pool of all potentially relevant tree species was assembled using the following criteria: (1) The species is a deciduous angiosperm that is native to Germany (to avoid strong effects of differences between angiosperms and gymnosperms); (2) the species is adapted to the site conditions including the ability to tolerate high light exposure at a young age and shade by fast-growing neighboring trees when older; (3) the selected species are widely spread across the angiosperm phylogeny (only one species per genus); and (4) species are either of economical or recreational relevance in Germany. The tree species that met these criteria were separated into two groups: one that usually associates with AMF and one that usually associates with EMF (based on a thorough literature research in 2014, and the comprehensive review by Wang and Qiu [2006]).

To select five species within each mycorrhizal group that were most similar according to plant functional traits (other than mycorrhizal association) between the mycorrhizal groups, we calculated functional diversity of the species in the two pools using the quadratic diversity Q index (Rao 1982; Appendix S1: Fig. S3). This minimized trait differences other than mycorrhizal type between AMF- and EMF-species pools that may confound effects of mycorrhizal type on ecosystem functioning. For the analysis, we used aboveground plant traits that are common but also represent plant growth rates, such as leaf out date, specific leaf area, maximum tree height, wood density, leaf C:N ratio, and seed mass. Growth rates are known to differ between AMF- and EMF-tree species. Our aim was to keep the number of species that follow this pattern at a minimum. The selected species are given in Table 1. We screened mycorrhization rates of all ten tree species in an 18-month pilot study with the similar design at the same site. All tree species were colonized by the respective predicted mycorrhizal type.

### Experimental design

We used a pool of five AMF- and five EMF-tree species in total (Table 1). This led to a manageable number of replicated monoculture plots (N = 20 plots; ten tree species with two replicates each), a comprehensive set of possible two-species combinations (N = 30 plots), and five replicates of different species compositions in the four-species mixtures (N = 30 plots). We established two replicated monoculture plots per species (Fig. 2c; Appendix S1: Fig. S5). Communities of two species were replicated ten times in total using different tree mixtures, but specific species compositions were not replicated. This allows quantification of species diversity effects that are not confounded by the effects of species identity or community composition. In the two-species mixtures with only AMF-trees and only EMF-trees, respectively, all possible species combinations were established (ten plots in total for each). In the two-species mixtures with both mycorrhizal types, only a subset of ten out of 25 possible combinations was established. In the four-species mixtures with only AMF-trees and only EMF-trees, respectively, all possible species combinations were implemented and each species composition was replicated twice. In the four-species mixtures with both mycorrhizal types, only a subset of ten out of 100 possible combinations was implemented. Subsets were chosen to keep an equal occurrence of all species across all experimental plots. Due to logistic and practical constraints, it was not possible to establish all possible species combinations or to fully balance the proportion of represented species combinations among all treatment levels. However, this was done in the 18-month pilot study mentioned above. In total, 80 plots were established in two blocks (Fig. 2b). Within each block, spatial arrangement of plots was random following the two preconditions that plots of the same treatment are not adjacent to each other and that in all eight plots directly surrounding the focal plot, the same treatment appears at most twice. Grass paths (3.5 m wide) were established between the plots.

The plots have a size of 121 m^2^ (11 × 11 m) with a 1.5 m buffer consisting of the outermost tree rows and a core area of 8 × 8 m (Fig. 2d). Samples and measurements are exclusively taken in the core area to reduce edge effects. In three plots, wireless data loggers measure year-round temperature and humidity in 1 m above soil surface and 5, 10, and 55 cm below soil surface at 30-min intervals.

Trees were planted at a distance of 1 m as a compromise between capturing early below-ground interactions between tree species and to minimize mortality of slow-growing species due to asymmetric competition for light with fast-growing species close-by. Trees were planted in a regular pattern (Fig. 2d) to mix species to the greatest extent possible. This fosters small-scale interactions belowground that may play a key role for small-sized organisms, such as soil invertebrates and microorganisms. The planting pattern also enables thinning at later stages. We planted 140 tree individuals per plot, which adds up to 11,200 trees in total (for details on site preparation and establishment of the experiment, see Appendix S1: Methods S1).

### Baseline measurements

After establishment of the experimental plots, a baseline soil sampling campaign and tree measurements were conducted after the first growing season (Appendix S1: Figs. S1 and S2). Subsequently, most soil and tree measurements have been and will be repeated annually to create time-series data that will allow us to assess whether temporal dynamics of ecosystem functions are affected by the experimental treatments.

MyDiv tests the influence of tree species and mycorrhizal type on a variety of belowground ecosystem functions and processes. Measurements of microbial properties, such as soil microbial biomass and basal respiration as well as community structure, are essential response variables. They are measured annually and biannually, respectively, because microorganisms represent the most abundant organism group in soil that drives many essential soil processes (van der Heijden et al. 2008). Other biological, chemical, and physical soil variables, such as soil pH, soil texture, and carbon, nitrogen, and phosphorus concentrations, are further important baseline measurements that are done biennially. Tree height, diameter at breast and 5 cm height, vitality, and damage within the community are assessed in all 64 tree individuals inside each plot core area on all plots annually. Additionally, a soil core of 1 m depth was taken from each plot and subdivided into eight layers (0–5, 5–10, 10–20, 20–30, 30–40, 40–50, 50–60, and 60–100 cm depth) and archived. During the first growing season, we started a time-series study on wood decomposition in the top soil by measuring mass loss of wooden tongue depressors buried over a period for six months (Baker et al. 2001).

### Integration of other projects and networks

Phytometers are a common tool in BEF experiments to assess plant community effects on focal plant physiology and morphology and related changes of multitrophic interactions in a highly standardized way (e.g., Gibson 2002, Scherber et al. 2006, Eisenhauer et al. 2009). In MyDiv, we go one step further in standardizing the phytometer approach by using in vitro plants of the oak clone (*Quercus robur* L.) DF159 (Herrmann et al. 1998) characterized at the molecular level by a de-novo transcriptomic reference bank (Tarkka et al. 2013). In the framework of the TrophinOak project (www.TrophinOak.de), this reference contig bank was used to characterize the oak phytometer DF159 at the molecular level and to analyze gene regulation during multitrophic interactions in relation to the rhythmic growth patterns displayed by *Q. robur* (Angay et al. 2014, Caravaca et al. 2015, Herrmann et al. 2015, 2016, Maboreke et al. 2017). Such phytometers (called PhytOakmeter) can help to deepen our understanding of mechanisms up to molecular level contributing to patterns on ecosystem level we find. Three PhytOakmeters were added to the plot core area of plots containing *Quercus petraea* (Matt.) Liebl. (monocultures, two-species, and four-species mixtures; 20 plots in total) in order to follow the EMF-mycorrhization status of the PhytOakmeter in relation to the molecular responses during the successive tree growth cycles during the entire growing season.

MyDiv is further an official member of the global collaborative network of tree diversity experiments called TreeDivNet (www.treedivnet.ugent.be; Verheyen et al. 2016). Experimental designs within this network share important characteristics, such as the manipulation of tree diversity in the field, separation of tree diversity from identity effects, a diversity gradient of at least three levels, and the assessment of multiple ecosystem functions. Cross experiment comparisons will provide tests of tree diversity effects on ecosystem functioning in different environmental contexts and will enable local truths to be separated from general patterns in BEF relationships (Grossman et al. 2018). The network currently consists of 25 experiments comprised of >1,100,000 trees across four biomes (Paquette et al. 2018).

### First results on tree diversity effects in relation to mycorrhizal type

**Fig. 3:**
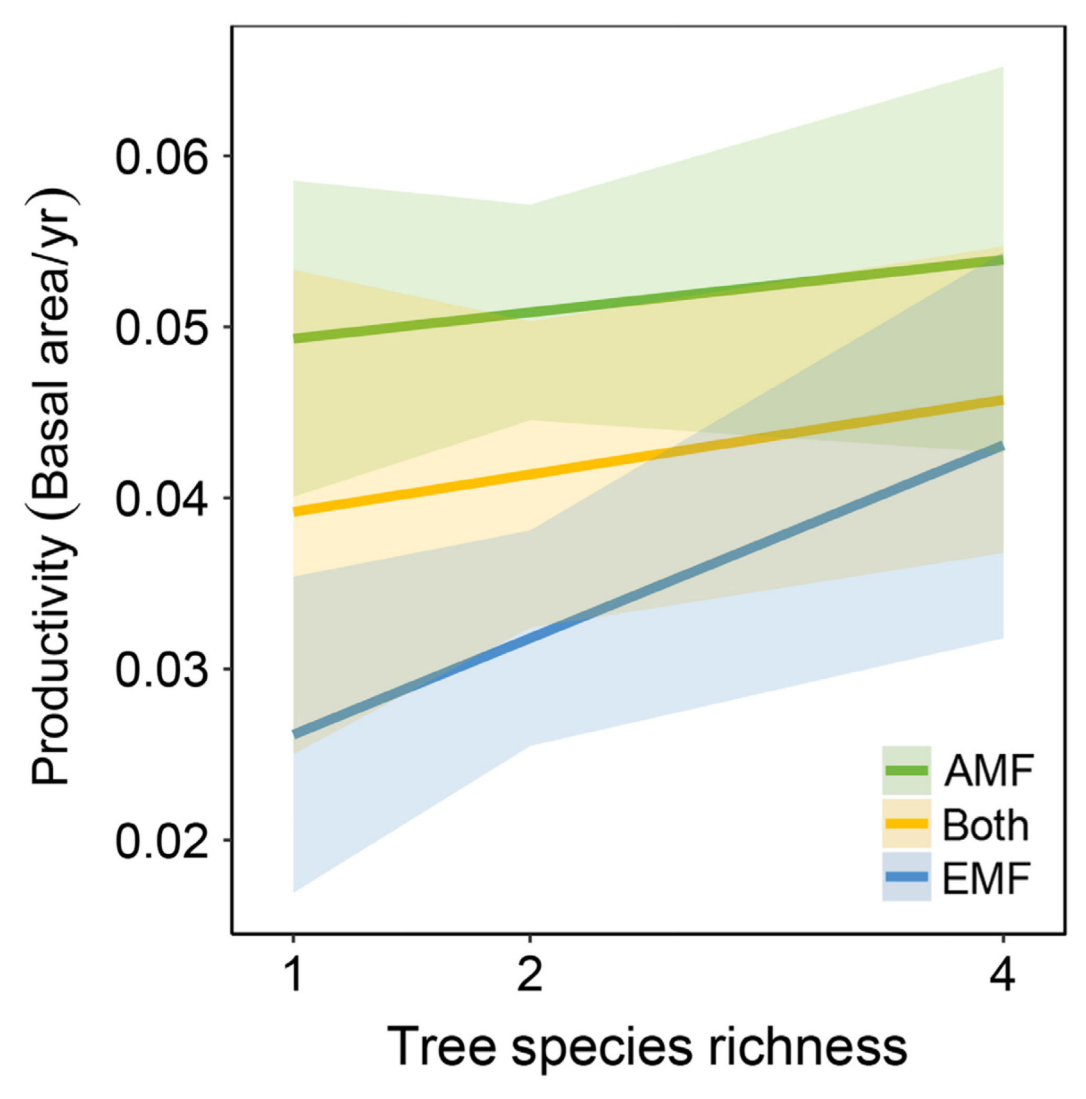
Tree productivity (March 2015–November 2017) in tree communities associated with only arbuscular mycorrhizal fungi (AMF), only ectomycorrhizal fungi (EMF), or communities with AMF- and EMF-trees in mixture as affected by tree species richness. Lines are estimated using a linear mixed-effect model. Colored bands represent 95% confidence intervals.

We calculated tree productivity as mean annual increment, that is, change in basal area (m^2^, where tree diameter was measured at 5 cm above the soil surface), between years one and two of the experiment (twenty months after establishment). We then calculated net biodiversity, complementarity, and selection effects following Loreau and Hector (2001) to separate effects of the dominance of highly productive single species from species richness effects on productivity (see Appendix S1: Methods S2, for details on the methods). Productivity varied significantly among mycorrhizal types (*F*_2,51.4_ = 3.90, *P* = 0.03) and increased marginally with tree species richness (*F*_1,50.2_ = 3.49, *P* = 0.07), but there was no significant interaction between the two (*F*_2,49.5_ = 0.63, *P* > 0.10; Fig. 3). On average, productivity was highest in communities of tree species associated with AMF. This was followed by communities containing a mixture of tree species associated with either AMF or EMF, and communities with tree species associated only with EMF (Fig. 3). While productivity was higher in tree communities with greater species richness, accounting for community composition as a random factor increased the amount of explained variation from 31% (marginal *R*^2^) to 84% (conditional *R*^2^).

**Fig. 4:**
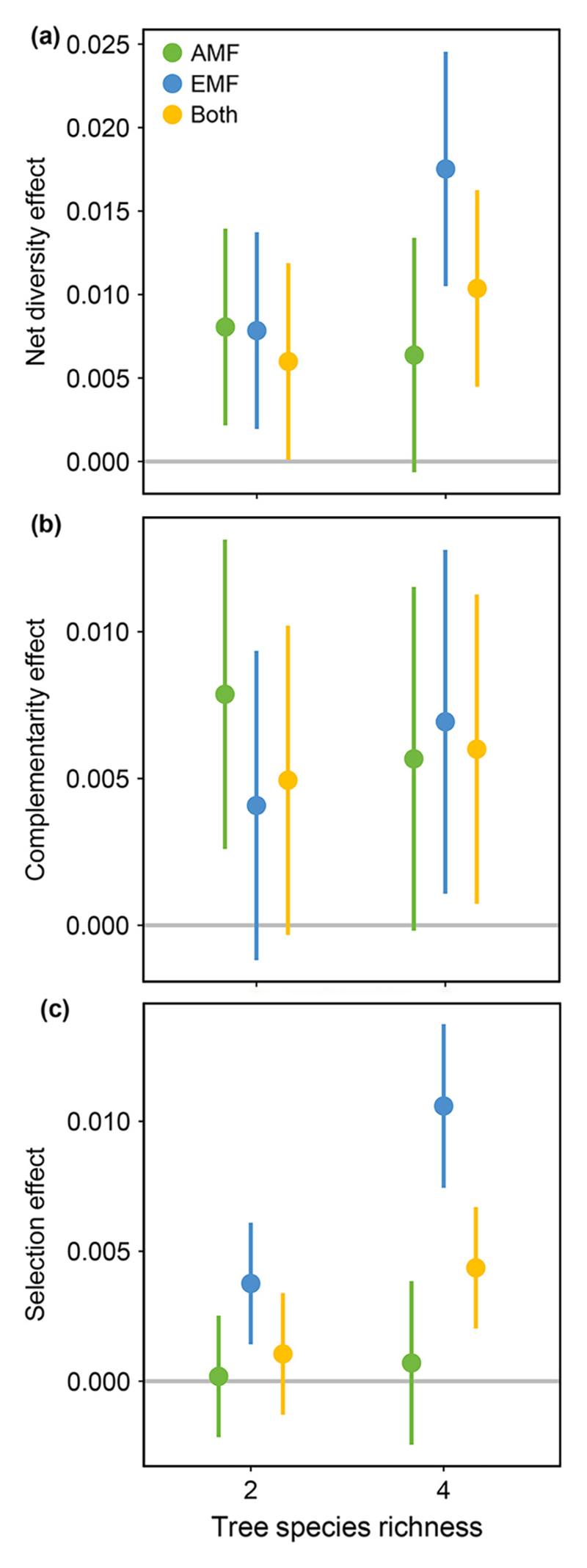
Coefficient estimates of linear mixed effects models for (a) net diversity, (b) complementarity, and (c) selection effects in tree communities associated with only arbuscular mycorrhizal fungi (AMF), only ectomycorrhizal fungi (EMF), or communities with AMF- and EMF-trees in mixture as affected by tree species richness. Whisker bars are 95% confidence intervals.

Two-species tree communities comprised of tree species associated with AMF had positive net biodiversity and complementarity effects (Fig. 4a, b; 95% confidence intervals do not overlap with zero) and that associated with EMF positive net biodiversity and selection effects (Fig. 4a, c). High diversity tree communities comprised of tree species associated with either EMF or both mycorrhizal types had positive net biodiversity, complementarity, and selection effects (Fig. 4a–c). However, net biodiversity effects and complementarity effects did not change with increasing (*F*_2,36.6_ = 1.66, *P* > 0.10; *F*_2,35.8_ = 0.14, *P* > 0.10), or with the interaction of the two (*F*_2,36.6_ = 1.55, *P* > 0.10; *F*_2,35.8_ = 0.43, *P* > 0.10), thereby indicating complementarity among species being of minor importance in the short term. In contrast, selection effects increased significantly with tree species richness (*F*_1,40.9_ = 10.96, *P* = 0.002) and differed among mycorrhizal treatments (*F*_2,41.0_ = 12.37, *P* < 0.001), but not with the interaction between the two (*F*_2,41.0_ = 2.62, *P* = 0.08). Selection effects were highest in tree species associated with EMF in four-species mixtures.

## Conclusions and Outlook

During the first two years of the MyDiv experiment, tree community productivity increased marginally significantly with tree species richness. However, the most productive tree communities were not, as hypothesized, the ones with both mycorrhizal types, but rather those that associate with AMF only. This result may be due to the fast growth of most of the AMF species. The strongest influence of tree diversity on tree productivity was observed in the EMF-communities and slightly weaker effects in mixed communities. The observed increases in productivity with tree diversity were due to the inclusion of highly productive species, primarily *Betula pendula* Roth (EMF), *Tilia platyphyllos* Scop. (EMF), and *Prunus avium* (L.) L. (AMF). Consequently, we found that selection effects drove early biodiversity effects in the EMF-communities. This is consistent with experiments in grasslands (e.g., Marquard et al. 2009) and forests (e.g., Tobner et al. 2014) showing that selection effects are more important than complementarity effects in early stages of experiments. Complementarity effects may become more important with time (Fargione et al. 2007, Reich et al. 2012). We speculate that these strong selection effects in EMF-communities may be explained by differences in life histories of the EMF-species (e.g., growth rates; Brzeziecki and Kienast 1994). Thus, the presented results have to be interpreted with caution, as the magnitude and mechanisms driving biodiversity effects on ecosystem functioning in MyDiv will likely change over time (Guerrero-Ramirez et al. 2017) as mycorrhiza colonize and continue to develop their hyphal networks. The long-term perspective of the experiment may shed light on processes underlying those temporal dynamics of biotic interactions.

MyDiv addresses the need to integrate biotic interactions, realized in the form of mycorrhizal types, into the experimental design of BEF experiments. MyDiv is one of the first experiments that focuses on the effects of identity and diversity of mycorrhizal types that typically co-occur in forest ecosystems and mediate resource use complementarity. To better understand the processes behind resource uptake strategies in the two mycorrhizal types and to test the predicted conceptual framework presented in Fig. 1, the future use of resource tracer experiments may be particularly promising (Gockele et al. 2014). MyDiv offers the opportunity for further subplot treatments to explore the basis of resource use complementarity. For instance, addition of different nutrients (e.g., combinations with nitrogen and phosphorus additions) and stable isotope tracers could further illuminate the role of specific groups of mycorrhizal fungi in nutrient uptake.

Furthermore, the long-term perspective of this tree diversity experiment allows for studying temporal dynamics in the contribution of mycorrhizal type identity and diversity to resource use among plants. In addition, it allows scaling up of species interactions and physiological processes from individuals to neighborhoods to plot-level ecosystem functions. For instance, the use of PhytOakmeters helps in conducting such measurements in a highly standardized way, but also all other tree species are well replicated along the diversity gradient. The PhytOakmeters combine the advantages of a laboratory study system, which to date is commonly used in studies with mycorrhiza and controlled multitrophic interactions (Herrmann et al. 2016), with the necessity to study biotic interactions with plants in more natural ecosystems, such as tree plantations where they interact with their biotic and abiotic environment. In future studies, a second clone may be introduced, for instance, a tree species associating with AMF. Thus, MyDiv implemented functional characteristics of plants in an experimental design to provide a conceptual framework for predicting resource use complementarity by considering biotic interactions with mycorrhizal fungi.

## Supplementary Material

Additional Supporting Information may be found online at: http://onlinelibrary.wiley.com/doi/10.1002/ecs2.2226/full

Supplementary Data

## Figures and Tables

**Table 1:** Tree species used in MyDiv with respective mycorrhizal type as reported in the literature.

Species	Family	Mycorrhizal type	References
*Acer pseudoplatanus* L.	Sapindaceae	AMF	Harley and Harley (1987a, b), Pirazzi et al. (1999), Weber and Claus (2000), Lang et al. (2011), Ruckli et al. (2014)
*Aesculus hippocastanum* L.	Sapindaceae	AMF	Harley and Harley (1987*a*), Bainard et al. (2011), Karliński et al. (2014)
*Fraxinus excelsior* L.	Oleaceae	AMF	Harley and Harley (1987*a*), Pirazzi et al. (1999), Weber and Claus (2000), Cesarz et al. (2013), Beyer et al. (2013), Kubisch et al. (2015)
*Prunus avium* (L.) L.	Rosaceae	AMF	Harley and Harley (1987*a*), Pirazzi et al. (1999), Aka-Kacar et al. (2010)
*Sorbus aucuparia* L.	Rosaceae	AMF	Harley and Harley (1987*a*), Sýkorová et al. (2016)
*Betula pendula* Roth	Betulaceae	EMF	Harley and Harley (1987*a*, *b*), Cuvelier (1990), Brun et al. (1995), Wright et al. (2005), Brundrett (2009)
*Carpinus betulus* L.	Betulaceae	EMF	Harley and Harley (1987*a*, *b*), Selosse et al. (2002), Brundrett (2009), Lang et al. (2011), Rewald et al. (2014)
*Fagus sylvatica* L.	Fagaceae	EMF	Harley and Harley (1987*a*, *b*), Selosse et al. (2002), Brundrett (2009), Beyer et al. (2013)
*Quercus petraea* (Matt.) Liebl.	Fagaceae	EMF	Harley and Harley (1987*a*, *b*), Bakker et al. (2000), Urban et al. (2008), Brundrett (2009)
*Tilia platyphyllos* Scop.	Malvaceae	EMF	Harley and Harley (1987*a*), Sisti et al. (2003), Brundrett (2009), Lang et al. (2011)

*Note:* AMF, arbuscular mycorrhizal fungi; EMF, ectomycorrhizal fungi.
