# Supplementary material for: Mycorrhiza in tree diversity–ecosystem function relationships: conceptual framework and experimental implementation
Source: Ecosphere. Author manuscript; Available in PMC 2018 Oct 13. (PMC6186167; doi:10.1002/ecs2.2226)
Supplement: Supplementary Data [file NIHMS80005-supplement-Supplementary_Data.pdf]

Ecosphere

Mycorrhiza in tree diversity-ecosystem function relationships: conceptual framework and experimental implementation

Olga Ferlian, Simone Cesarz, Dylan Craven, Jes Hines, Kathryn E. Barry, Helge Bruelheide, François Buscot, Sylvia Haider, Heike Heklau, Sylvie Herrmann, Paul Kühn, Ulrich Pruschitzki, Martin Schädler, Cameron Wagg, Alexandra Weigelt, Tesfaye Wubet and Nico Eisenhauer

Appendix S1

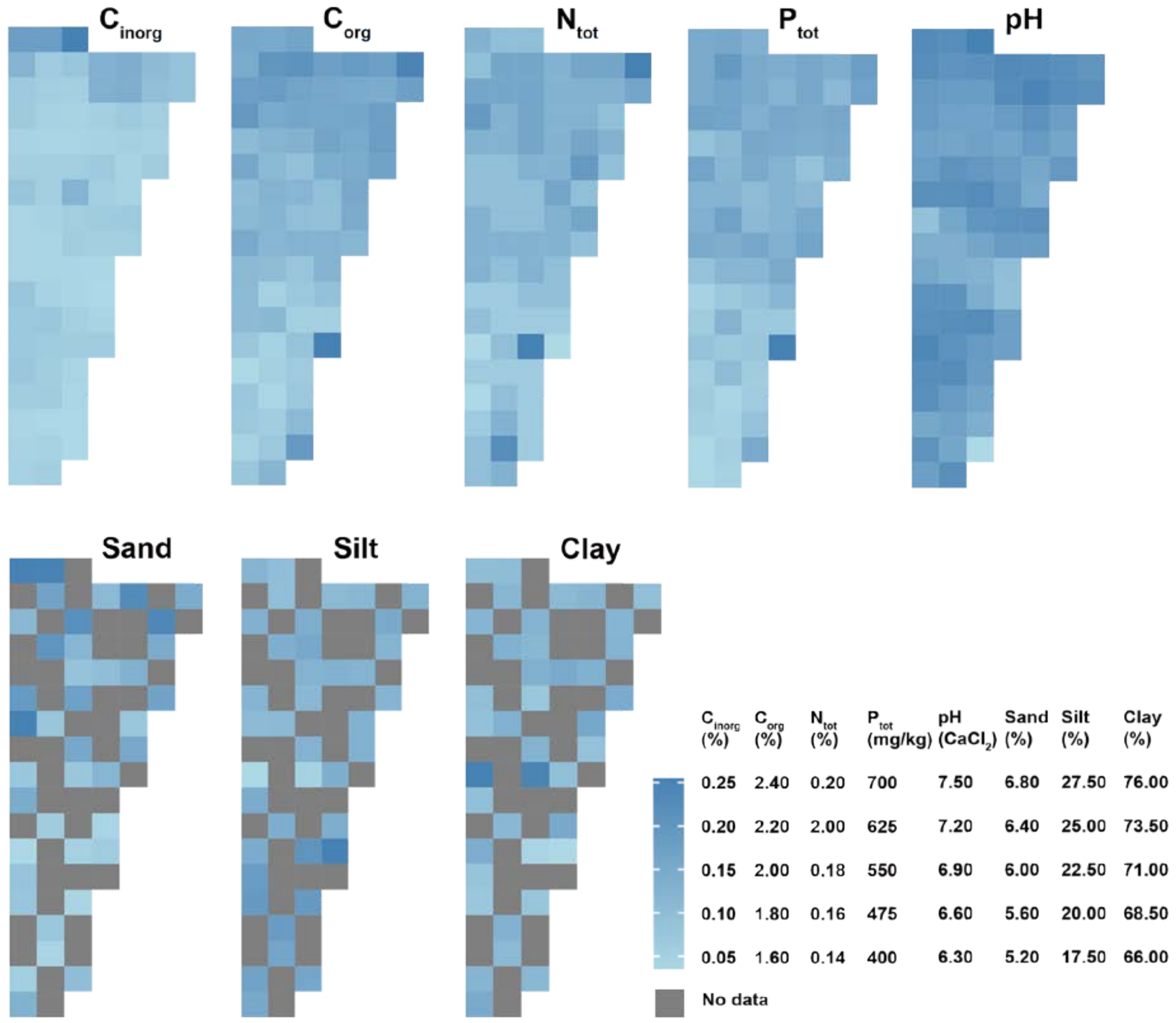

Figure S1 Heatmaps of abiotic parameters (C_{inorg}: inorganic carbon, C_{org}: organic carbon, N_{tot}: total nitrogen concentrations, P_{tot}: total phosphorus concentrations, soil pH, and soil texture) measured in MyDiv six months after establishment. Grey bars represent plots without data on soil texture.

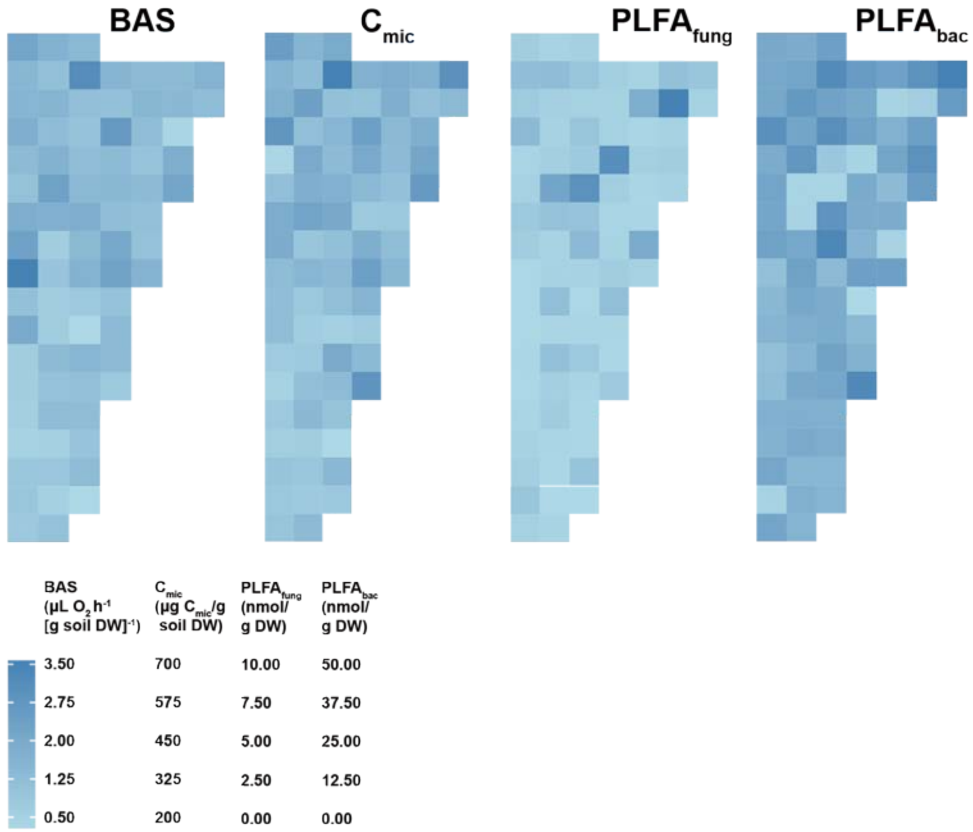

Figure S2 Heatmaps of microbial parameters (BAS: microbial basal respiration, C_{mic}: microbial biomass carbon, $PLFA_{fung}$: concentrations of fungal PLFAs, $PLFA_{bac}$: concentrations of bacterial PLFAs; concentrations of fungal and bacterial PLFAs are a measure of fungal and bacterial biomass after Frostegård & Bååth (1996)) measured in MyDiv six months after establishment.

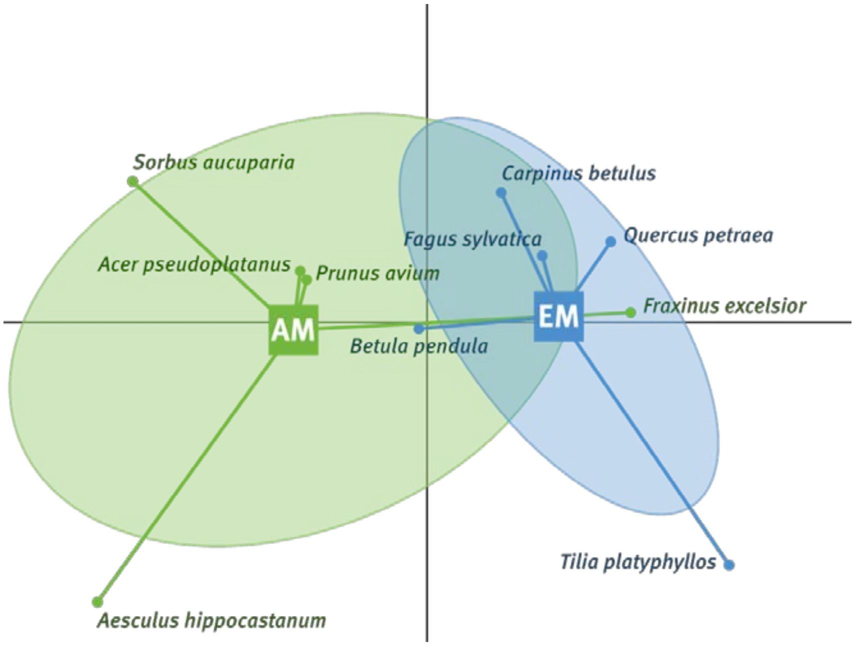

Figure S3 Principal coordinates analysis of selected tree species in MyDiv illustrating interspecific differences in the following functional traits: tree height, wood density, specific leaf area, seed mass, leaf C:N ratio, and leaf out date. Tree species with arbuscular mycorrhizal fungi in green (AM), species with ectomycorrhizal fungi in blue (EM).

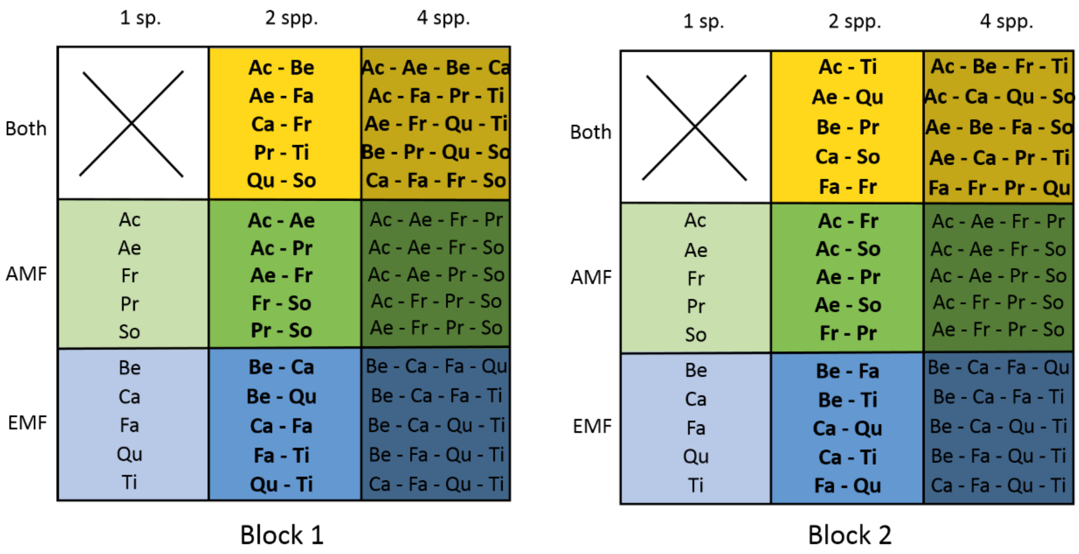

Figure S4 Species combinations for each treatment in the two blocks of MyDiv. Treatments with letters in bold indicate different species combinations in the two blocks, whereas the other species combinations are replicated in the blocks. AMF: arbuscular mycorrhizal fungi, EMF: ectomycorrhizal fungi, Both: both mycorrhizal types.

Methods S1 Preparation and establishment of MyDiv

In preparation for tree planting, the site was ploughed to a depth of 30 cm and subsequently grubbed in January and February 2015. After delimiting plot squares, planting positions for all tree seedlings were marked and one- to two-year old seedlings (depending on species, 50 - 80 cm in height) were planted. Only individuals of the regional provenance were used. Seedlings had been cultivated by a commercial nursery in arable soil including yearly NPK-fertilisation treatments. In March 2015, they were planted bare-rooted into holes made by a motor-driven soil corer. Planting was finished within three days and spare seedlings were planted in a separate area for replacement of dead seedlings in the plots. The soil of each plot was covered with black, water-permeable weed tarp (1 m width) that was secured with metal pegs in the middle and buried at the edges. The tarp shall minimise weed growth and, thus, competition with seedlings as well as the establishment of mycorrhizal fungi associated with other plants than tree seedlings. The tarp will be removed as

soon as a closed tree canopy develops. The whole site was enclosed by a game fence to prevent damage of young trees by deer and hare. Several live traps and wildlife cameras were set up to monitor activity of voles, other mammal herbivores, and potential vole predators at the site.

Trees were watered in summer 2015 to minimise mortality during the dry season of the first year. Tree survival is assessed twice a year, shortly after leafing out and shortly before leaf fall. Survival rate after the first growing season was 95.9% across all species and treatments. However, survival rates varied among species. The lowest survival rates were found for *Q. petraea* (81.9%) and *F. sylvatica* (85.2%), which was presumably due to cutting of seedling roots by the nursery and the intolerance of direct radiation in the latter species. All other species had survival rates higher than 97.8%. Trees were replanted after each survival assessment in the first two years. Weeds are removed from plots, every year in summer. The grass paths of the site are mown regularly and will be ploughed and resown once every few years in the future to disrupt hyphal networks between plots that may interconnect belowground.

Methods S2 Calculations of biodiversity effects in MyDiv

Following Loreau and Hector (2001), the net diversity effect is the difference between the observed and expected productivity in a mixture, where the latter is the average of the monoculture biomass of the component species. The net diversity effect can be partitioned into complementarity and selection effects. The complementarity effect represents the productivity of a species in a mixture relative to its productivity in monoculture and is calculated as: N x M x ΔRY, where N is the number of species, M is the average productivity of a species in a monoculture and ΔRY is (Y/ M) - 1/N. The selection effect is N x cov(M,ΔRY) and reflects whether species with high yields in monocultures also dominate in mixtures.

We fitted separate linear mixed-effects models that test for the effects of tree species richness, mycorrhizal group, and the interaction of the two on productivity, net biodiversity, complementarity, and selection effects. Experimental block was also included as a fixed effect to remove its potential contributions explained by other fixed effects and a species composition factor was used as a random group effect (Schmid et al., 2017). Tree species richness was treated as a continuous variable for productivity but as a categorical variable for net biodiversity, complementarity, and selection effects. Models were fitted in R (R Core team 2014) using the

‘lmer’ function in the ‘lme4’ package (Bates et al., 2009) and visualised using ‘ggeffects’ (Lüdecke, 2018) and ‘ggplot’ (Wickham, 2009).

Literature Cited

Bates D., M. Maechler, B. Dai. 2009. lme4: Linear mixed-effects models using S4 classes. 2009. R package version 0.999375-31. URL: http://CRAN.R-project.org/package= lme4.

Frostegård, Å., E. Bååth. 1996. The use of phospholipid fatty acid analysis to estimate bacterial and fungal biomass in soil. Biology and Fertility of Soils 22:59-65.

Lüdecke D. 2018. ggeffects: Create Tidy Data Frames of Marginal Effects for 'ggplot' from Model Outputs. R package version 0.2.2. URL: https://CRAN.R-project.org/package=ggeffects.

Loreau M., A. Hector. 2001. Partitioning selection and complementarity in biodiversity experiments. Nature 412:72-76.

R Core Team, 2014. R: A Language and Environment for Statistical Computing. R Foundation for Statistical Computing, Vienna, Austria. https://www.R-project.org/.

Schmid B., M. Baruffol, Z. Wang, P. A. Niklaus. 2017. A guide to analyzing biodiversity experiments. Journal of Plant Ecology 10:91-110.

Wickham H. 2009. ggplot2: Elegant graphics for data analysis. Springer, New York. URL: http://ggplot2.org.
